# Molecular Characterization and Risk Factors of* Giardia duodenalis* among School Children from La Habana, Cuba

**DOI:** 10.1155/2015/378643

**Published:** 2015-11-29

**Authors:** Luis Enrique Jerez Puebla, Fidel A. Núñez, Isabel Martínez Silva, Lázara Rojas Rivero, Marta Martínez González, Yuliet Méndez Sutil, Lucía Ayllón Valdés, Iraís Atencio Millán, Norbert Müller

**Affiliations:** ^1^Department of Parasitology, Tropical Medicine Institute “Pedro Kourí”, Autopista Novia del Mediodía Km 6^1/2^ e/Autopista Nacional y Carretera Central, Habana, Cuba; ^2^Paediatric Hospital “William Soler”, Avenida 100 y Perla, Altahabana, Boyeros, Habana, Cuba; ^3^Institute of Parasitology, Vetsuisse Faculty, University of Bern, Bern, Switzerland

## Abstract

*Giardia duodenalis* is considered the most common protozoan infecting humans worldwide. Molecular characterization of* G. duodenalis* isolates has revealed the existence of eight groups (assemblages A to H) which differ in their host distribution. A cross-sectional study was conducted in 639 children from La Habana between January and December 2013. Two assemblage-specific PCRs were carried out for the molecular characterization. The overall prevalence of* Giardia* infection was 11.9%. DNA from 63 of 76 (82.9%) samples was successfully amplified by PCR-*tpi*, while 58 from 76 (76.3%) were detected by PCRE1-HF. Similar results by both PCRs were obtained in 54 from 76 samples (71%). According to these analyses, assemblage B and mixed assemblages A + B account for most of the* Giardia* infections in the cohort of children tested. Our current study identified assemblage B as predominant genotype in children infected with* Giardia*. Univariate analysis indicated that omission of washing hands before eating and keeping dogs at home were significant risk factors for a* Giardia* infection. In the future, novel molecular tools for a better discrimination of assemblages at the subassemblages level are needed to verify possible correlations between* Giardia* genotypes and symptomatology of giardiasis.

## 1. Introduction

*Giardia duodenalis* is an important cause of diarrhea in humans worldwide, especially in children, and is responsible for an estimated 2.0 million cases per year [1]. The prevalence of this infection is higher in developing countries as the poor sanitary conditions favour the contamination of water and food with cysts. This parasite has been included in the Neglected Diseases Initiative of the WHO due to its diffusion among children in these regions of the world, and being a significant cause of diarrhea and nutritional disorders in institutional and community settings [2]. Approximately 200 million people have symptomatic giardiasis in Asia, Africa, and Latin America and about 500,000 new cases are reported each year according to the World Health Organization (WHO) [3].

Giardiasis exerts a significant public health impact because of the high prevalence and disease burden of the infection and its propensity in causing major outbreaks and emergency responses [1]. An important aspect of the epidemiology of giardiasis is to understand the host range of different* Giardia* species and strains/genotypes, the potential for cross-species transmission, and risk environmental factors involved in the exposure of the pathogen. This is particularly important in determining the human disease burden attributable to parasites of animal origin and in determining the zoonotic potential of* Giardia* infections in domestic animals [4].

*G. duodenalis*, which is found in humans and many other mammals, including pets and livestock, is now considered a multispecies complex that comprises at least eight distinct genetic groups, referred to as assemblages A to H [4]. The analysis of more than a thousand human isolates from different geographical locations, examined by PCR amplification of DNA extracted directly from stool, has demonstrated that, in almost all cases, only* G. duodenalis* assemblages A and B are associated with human infections [2]. In the course of these studies, it has been observed that the analysis of more than one gene is needed to address the occurrence of mixed infection in humans and the correlation between clinical symptoms and the type of* Giardia* assemblage [4].

The spectrum of clinical manifestations in human giardiasis is relatively variable, ranging from the absence of symptoms to acute or chronic diarrhea, dehydration, abdominal pain, nausea, vomiting, and weight loss [5]. Children might also suffer more serious consequences, including retarded growth and development, poor cognitive function, and detrimental effects on nutritional status [6].

With regard to that issue, studies on the correlation between the assemblages and clinical symptoms have reported controversial results. Some studies have pointed out that symptoms are more associated with assemblage A [7], while others have found that assemblage B infections [8] are more likely to be symptomatic. Recent studies made by Pelayo et al. (2008) and Puebla et al. (2014) in a group of children from La Habana have found that children harboring assemblage B of* Giardia* were more likely to have symptomatic infections than children with isolates from assemblage A [9, 10].

Giardiasis is an endemic intestinal parasitic infection in Cuba with prevalence rates ranging from 10 to 55% in the most recent studies, mainly in the capital and the western provinces of the country [11–13]. In the last national survey of intestinal parasitic infections (IPI) carried out in 2009,* Giardia duodenalis* was the most frequently identified pathogenic protozoan [14].

Since the identification of the genetical assemblage is considered a primary element in the study of human giardiasis, it is important to develop and apply, in laboratories with basic molecular equipment, single step PCR methods that allow one to detect and distinguish assemblages A and B and mixed infections from human faecal specimens simply by gel electrophoresis of the amplification products [15]. That is why the present study was conducted to explore whether there is an association between assemblages and clinical manifestations by the performance of two conventional PCR tests and to identify epidemiological risk factors associated with this infection.

## 2. Methods

### 2.1. Study Design and Study Population

A descriptive cross-sectional study was conducted on 639 children from 4 primary schools located in urban settings belonging to the Plaza de la Revolución, Playa, and Boyeros municipalities, in La Habana. They were subjected to a routine examination as part of a program of intestinal parasitic surveillance, from January 2013 to December 2013. In this work, we studied the frequency of* G. duodenalis* in children from preschool up to 4th grade from each school. The inclusion criterion for this study was all stool samples positive to* G. duodenalis*. Cases in which coinfection with other intestinal parasites was found were not included in the investigation between infecting assemblages and clinical signs.

### 2.2. Collection of Stool Samples

Stool collection containers were handed to the parents who were instructed to bring a fresh sample from the child on the day of examination. Three stool samples from each child involved in this study were examined for intestinal parasites. A standard questionnaire was used to collect information on each child based on the demographic (e.g., age, sex, and number of household members), environmental (e.g., availability and types of toilets in the household, types of water supply, garbage disposal, and presence of domestic animals), personal hygiene (e.g., washing hands before eating, after defecation, and after playing with animals, washing vegetables and fruits before consumption, boiling water before consumption, and bathing place), and general health status of the participants (i.e., symptoms related to intestinal parasitic infections such as diarrhoea (defined according to WHO (1998)), nausea, vomiting, and abdominal pain and a history of receiving anthelmintic treatment). Symptomatic children were defined as those children who had at least one of the symptoms mentioned above and included in the questionnaire, while the asymptomatic children were referred to the complete absence of symptoms.

### 2.3. Processing of Samples

Fresh fecal samples were collected in a labeled and sterile fecal container. A portion of stool was examined at field by a wet smear stained with Lugol's iodine and followed by formalin ethyl acetate concentration techniques under light microscope at 10x and 40x magnifications. All the diarrheal stool samples were stained by modified acid-fast for* Cryptosporidium* spp.,* Cyclospora*, and* Cystoisospora* [16]. The Kato-Katz smear method was used for species specific identification of parasite eggs, including* Ascaris lumbricoides*,* Trichuris trichiura,* and hookworm. All samples were tested the same day that they were collected. All* Giardia*-positive samples were stored in potassium dichromate (2.5%) and stored at −20°C for molecular characterization.

### 2.4. Purification of* Giardia duodenalis* Cysts and DNA Extraction

*Giardia* cysts were purified and concentrated from stool samples in a sucrose gradient with a specific gravity of 0.85 M and then washed with distilled water, following the protocol described by Babaei et al. [17]. The cyst wall was disrupted by 8–10 freeze-thaw cycles in liquid nitrogen alternated with a 95°C water bath. After that, purified cysts were mixed with 300 *μ*L of buffer lysis (50 mM Tris-HCl, pH 7.5; 25 mM EDTA, 25 mM NaCl, and 1% of sodium dodecyl sulphate (SDS)) and vortexed. After adding 100 *μ*g/mL of proteinase K, the suspension was incubated at 56°C for 2 h.

The DNA lysate was then treated with phenol/chloroform/isoamyl alcohol (24 : 24 : 1), followed by chloroform/isoamyl alcohol (24 : 1) according to Sambrook and Russell protocol (2001) [18]. The DNA was precipitated by the addition of 1 mL chilled ethanol and stored at −20°C until use. The dried DNA was resuspended in 30 *μ*L distilled water and used as a template for PCR.

### 2.5. Molecular Analysis

DNA molecular analysis was carried out using published PCR-relevant protocols [15, 19]. In brief, the genotyping analysis was performed using assemblages-specific primers that amplify, in the case of the PCR-*tpi*, a 148-bp fragment of the assemblage A and 81-bp fragment of assemblage B [19]. The other PCR (PCR-*E1-HP*) amplifies a 165-bp amplicon for assemblage A and 272-bp fragment for assemblage B [15].

The PCR reaction mixture was done using a AmpliTaq DNA Polymerase with GeneAmp 10x PCR Buffer Kit (Applied Biosystems, USA) in a total volume of 25 *μ*L and comprised 10 *μ*L of 10x PCR buffer (Applied Biosystems, USA), 0.2 mM of each deoxynucleoside triphosphate (dNTP) (Applied Biosystems, USA), 1 U of Taq polymerase (Applied Biosystems, USA), 0.4 *μ*M of each primer, and 5 *μ*L of DNA template, with ultrapure water used as a negative control.

The DNA was amplified using a thermocycler (Gene Amp PCR System 9700, Applied Biosystems, USA). The PCR products were analyzed by 2% agarose gel electrophoresis, stained with 0.5 *μ*g/mL of ethidium bromide, and then visualized on a UV transilluminator (Syngene, U:Genius, Belgium).

DNA from axenic cultures of* G. duodenalis* strains WB-C6 (assemblage A) and GS (assemblage B) was used as positive controls, while ultrapure water was included in negative controls.

### 2.6. Statistical Analysis

All data were analyzed using EPINFO 6.04 and EPIDAT 3.1 statistical programmes. Chi square test and proportion tests were employed to assess the significance of the associations. Fisher's exact test was used when required due to data scarcity.

Univariate analyses used all available input variables. A multivariate logistic regression using introduction tests was used to determine whether each independent variable was significantly related to the outcome variable. The odds ratio (OR) with 95% confidence intervals (CI) was used to approximate the relative risk associated with exposure. The *P* values less than 0.05 were considered as statistically significant for all tests.

### 2.7. Ethical Considerations

The research protocol was approved by the Ethics Committee of the “Pedro Kourí” Institute. Written informed consent was obtained from parents/guardians for children to participate in the study. Each symptomatic child was seen and treated by a pediatric specialist.

## 3. Results

### 3.1. Characteristics of the Study Population

From a total of 639 children examined, 76 were infected with* G. duodenalis* for an overall prevalence of 11.9%. In two cases, coinfection with* Cryptosporidium* spp. and* Entamoeba histolytica*/*E. dispar* were recorded. From the 76* Giardia*-infected children included in the study, 41 (53.9%) were symptomatic and 35 (46.1%) did not present symptoms.

No significant association was found between* G. duodenalis* infection related to sex, residing area, age, or development of diarrhea between symptomatic and asymptomatic children (*P* > 0.05) (Table 1).

### 3.2. Combined Results from PCR-*tpi* and PCR*-E1-HP* PCR

**Figure 1:**
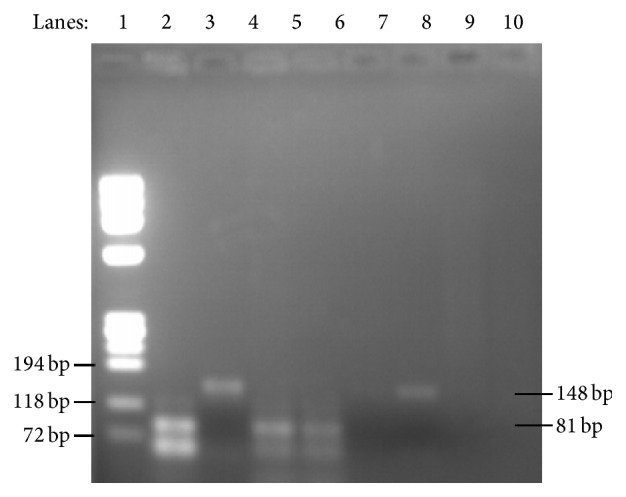
Ethidium bromide-stained 2% high-resolution agarose gel showing DNA amplified at the triosephosphate isomerase (*tpi*) gene. Lane 1, molecular marker Φ X174DNA/BsuRI (HaeIII); lane 2, positive control to assemblage B (81 bp); lane 3, positive control to assemblage A (148 bp); lanes 4-5, positive samples to assemblage B; lanes 6-7, positive samples to assemblage A; lanes 8-9, negative controls for both assemblages.

**Figure 2:**
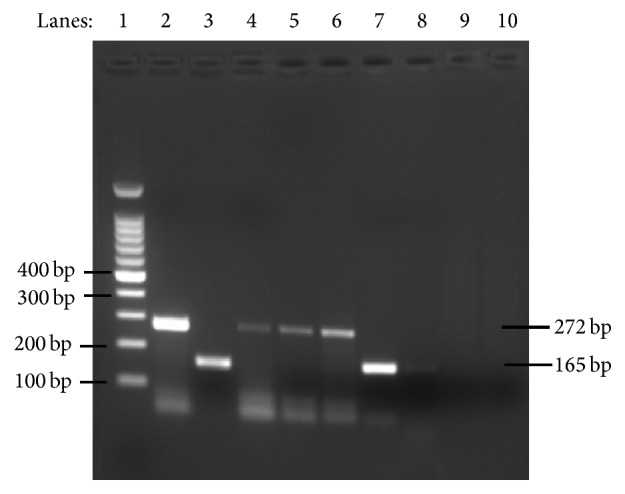
2% agarose gel showing PCR products specific for assemblages A and B according to Vanni et al., 2012. Lane 1, molecular marker 100 bp ladder; lane 2, positive control for assemblage B (272 bp); lane 3, positive control for assemblage A (165 bp); lanes 4-6, representative samples positive for assemblage B; lanes 7-8, representative samples positive for assemblage A; lanes 9-10, negative controls for assemblages A- and B-specific PCRs.

The 76 stool samples microscopically positive for* Giardia* were analyzed by PCR using two loci (*tpi* and* E1-HP*) as targets. From these samples, 63 (82.9%) and 58 (76.3%) scored positive in the PCR-*tpi* (Figure 1) and PCR-*E1-HP* (Figure 2), respectively, providing cumulated positivity of 85.5% (Table 2). In total, 43 positive samples (56.6%) revealed genotyping results that were confirmed by both PCRs. Conversely, 22 samples (28.9%) showed inconsistent genotyping results. Eleven samples (14.5%) scored negative in both PCRs, 10 of which showed very few cysts in the coprological examination.

*G. duodenalis* assemblage B was most frequently detected by both PCRs: 47.6% by PCR-*tpi*, and 67.3% by PCR-*E1-HP*. We detected only 7 cases (8.6%) of assemblage A-type infection confirmed by PCR-*tpi* and 5 cases (11.1%) by PCR*-E1-HP* only. Interestingly, mixed infections consisting of both assemblages were found in a relatively high proportion (41.3% by PCR-*tpi* and 24.1% by PCR*-E1-HP*, resp.).

### 3.3. Correlation between Assemblages and Symptoms

We also focused on the analysis of the clinical significance of* G. duodenalis* infection in children that harbored* Giardia* genotypes consistently identified in both PCRs (Table 3). Due to the low number of exclusive assemblage A-type infections detected in the child population, no statistical correlation between this particular assemblage and any symptom typical for giardiasis was possible. Diarrhea was the only clinical characteristic statistically associated with infection by assemblage B of* G. duodenalis* (*P* < 0.05) at least as compared to mixed A + B infections. For the other variables analyzed, no correlation was found between symptoms and infection with specific assemblages.

### 3.4. Risk Factors Associated with* Giardia duodenalis* Assemblages A and B Infections

Table 4 shows that univariate analysis reveals that omission of washing hands before eating (OR = 1.6, 95% CI: 1.00–2.64; *P* = 0.04) and keeping dogs at home (OR = 2.6, 95% CI: 1.23–5.36; *P* = 0.004) were significant risk factors for* Giardia* infection. Multivariate analysis using introduction test logistic regression ratified the association of contacts with dogs (OR = 2.1, 95% CI: 1.11–4.76; *P* = 0.01) with this intestinal infection.

## 4. Discussion

*Giardia duodenalis*, originally regarded as a commensal microorganism until 1978, is the etiologic agent of giardiasis, a gastrointestinal disease of humans and animals [1]. Giardiasis causes major public and veterinary health concerns worldwide. Transmission is either direct, through the faecal-oral route, or indirect, through ingestion of contaminated water or food. In developing,* Giardia* infection is acquired during early infancy and its prevalence peaks at up to 30% in children younger than 10 years of age. Apart from diarrhea, giardia infection in children in these countries can result in faltering of long-term growth and impairment of cognitive function [1, 20]. That is why it is important to assess the prevalence of this parasite in the child population, as well as the infecting assemblage, in order to elucidate the relationship between developed symptomatology and genetic groups of* Giardia* that could lead to changes and reduce the need for treatment of children.

Previous studies demonstrated* Giardia* as the principal intestinal protozoan that infects children around the country with prevalence rates reported from 10 to 55% [11–13]. In this investigation, a frequency of 11.9% was found in all children included. This result is lower when compared with a recent study conducted by Puebla et al. (2014) in symptomatic and asymptomatic children from La Habana, which showed an overall prevalence of giardiasis at 22% (*n* = 452). That confirms the endemism of this disease in younger children and the relatively high prevalence of* Giardia* infection in Cuban children.

The molecular characterization of* Giardia* from stool samples yielded the amplification of 63 of 76 samples (82.9%) by the* tpi* gene and 58 of 76 (76.3%) by the assemblage-specific PCR assay reported by Vanni et al. [15]. Here,* G. duodenalis* assemblage B parasites were preferentially detected in both PCRs (47.6% (PCR-*tpi*) and 67.2% (PCR-*E1-HP*), resp.). This observation is in accordance with most large-scale studies where the distribution of assemblage B was more commonly identified in developing (58%) than in developed countries (55%) and at a higher prevalence than assemblage A (37% versus 40%) [21].

In the present study, we found a high portion of mixed infections (41.3% by PCR-*tpi* and 24.1% by PCR*-E1-HP*, resp.; see Table 2) among the children studied. In a previous investigation, we found a frequency of mixed infections of 17.8% [10]. Recent studies using either conventional PCR with assemblage-specific primers [22] or real time PCR including sequencing of the resulting amplification products [23] showed a much larger degree of mixed assemblage infections in humans as previously reported [8, 24, 25].

The occurrence of mixed infections was also noted lately in some investigations using assemblage-specific* tpi* primers [26, 27], which allow detection of a much higher number of mixed assemblages A and B infections than approaches based on the use of more general primers for PCR [8, 25]. Other assemblage-specific PCRs developed by Vanni et al. (2012) can even detect individual assemblages when they are 9 times underrepresented in the sample in relation to assemblage B [15].

The reliable detection of cases of mixed infections is influenced by several factors, including the proportion of each assemblage in the specimen and biased amplification efficiencies of one assemblage over the other. In the present study, the use of the* tpi* gene for PCR was very important because it allowed detection of a higher number of mixed infections as compared to the PCRs designed by Vanni et al. (2012). Furthermore, the high variability of the* tpi* sequence (Geurden et al., 2008) makes the respective PCR an ideal molecular tool for a differential diagnosis of assemblage A-type versus assemblage B-type infections.

In the present study, inconsistent genotyping results in the PCRs applied were observed in 22 isolates (28.9%), representing a much higher number of such cases than previously reported (10 and 30%) [28, 29]. This discrepancy might be due to a bias of the PCR-*E1-H* towards a detection of assemblage B (see Table 2).

Molecular characterization of* G. duodenalis* is complicated by our incomplete understanding of the genetics of this organism. Many studies were based on the analysis of a single locus, particularly the 18S rDNA locus. Nowadays, through the application of multilocus PCR analysis there is a higher identification of mixed infections caused by assemblages A and B, and then inconsistent results can arise for assemblage (or even species) determination of given* Giardia* isolates [29].

Inconsistencies in genotyping results were observed in both human and animal (particularly dogs but also cattle) isolates of* G. duodenalis* [29] and can be formally explained by two distinct phenomena: (i) the presence of genetically different cysts in a faecal sample in combination with preferential amplification of a particular assemblage-specific marker gene compared to another marker gene indicative for a second assemblage (i.e., a true mixed infection followed by biased PCR amplification); or (ii) previous occurrence of sexual recombination among two* Giardia* assemblages that had led to a mixed genotype as far as the two PCR targets are concerned. Evidence of mixed infections with particularly high prevalences in infected individuals living in developing countries has been frequently provided [8, 30]. However, the second possibility also should not be underestimated because sexual genetic exchange between* Giardia* isolates has been previously (and is currently) discussed as a realistic scenario [31].

Differences in symptomatology of giardiasis with different assemblages were initially described for Dutch patients. Here, assemblage A was associated with mild intermittent disease but assemblage B was associated with severe persistent disease [32]. Since then, several studies have reported correlations between assemblages and symptoms, but there has been a lack of concordance in the data obtained [9, 32, 33]. However, these findings are contrasted by data that counterindicate a relationship between symptomatology and the infecting assemblage [33, 34]. Herein, we did a correlation analysis between* Giardia* assemblages and symptoms manifested in children. In the 43 children with identical genotyping results, we found that assemblage B was significantly more associated with diarrhea (*P* < 0.05) when compared with mixed infections by both assemblages. More data are needed to determine whether mixed infections could reduce symptoms induced by assemblage B.

In previous studies done in our country by Pelayo et al. (2008) and Puebla et al. (2014), a close association between assemblage B and symptomatic school children was found. However, the high proportion of mixed infections reported in both investigations further complicated the attribution of symptoms to infection with a specific assemblage. Compared to our previous studies [9, 10], our present data indicate a decline in the prevalence of assemblage B in symptomatic children although those studies had different overall prevalence rates of* Giardia* and the genetic characterization employed different markers. Nonetheless, assemblage B still has to be considered as the predominant assemblage within the* Giardia*-infected children in our country. Conversely, in this survey only a statistically insignificant number of assemblage A-type infections were found, thus excluding an association of this assemblage with a certain symptomatology.

The prevalence of* Giardia* is strongly associated with a variety of risk factors related to the host, such as sociodemographic, environmental, and zoonotic conditions [3]. Since water plays a major role in* Giardia* transmission [1], the quality of water is an essential parameter for risk factor assessment in giardiasis [35]. Apart from that, hygienic behaviour of children must be considered as a main risk factor of this disease. Surprisingly, however, most studies on hand hygiene related to intestinal parasites undertaken so far have not placed special emphasis on giardiasis [36]. Referring to the clinical significance of* G. duodenalis* among children in our country, we decided to investigate the abovementioned risk factors as well as various other epidemiological parameters regarding their relevance for transmission of this disease. Finally, omission of washing hands before eating and keeping dogs in-house turned out to be the only factors that were significantly associated with higher* Giardia* infection rates. These results are in agreement with recent findings in school children populations from developing Asian countries [37, 38].

The zoonotic transmission of* G. duodenalis* has gained increasing evidence, particularly as far as the role of domestic animals is concerned. Furthermore, it has been reported that dogs can harbor either zoonotic or canine-specific* Giardia* assemblages [4]. There is clear evidence that cysts of zoonotic* Giardia* do contaminate the environment in areas where the potential for zoonotic transmission exists. In the past, many epidemiological studies on giardiasis were able to identify a risk for zoonotic transmission of the disease. However, evidence of how frequently this phenomenon occurs requires intense focal studies in defined endemic areas where transmission dynamics and range of hosts involved in* Giardia* infections are known [4].

Considering the interest in unraveling the complex questions about the epidemiology of* G. duodenalis*, studies have focused on application of molecular methods to gain insight into genetic diversity of isolates and the public health significance of this, mainly in populations living in developing countries. The present study provides information on the* Giardia* assemblages associated with human infection and, more specifically, the association of epidemiological parameters with the infection of* Giardia* in school children. Further studies on the epidemiology of giardiasis, especially investigations of risk factors associated with* Giardia* assemblages, subassemblages, and genotypes, would help to better understand the characteristics of this disease. Such information will certainly be highly beneficial for the development of prevention and control strategies in giardiasis.

In conclusion, Giardiasis is one of the most frequent intestinal parasitic infections in Cuban children. Determination of* G. duodenalis* assemblages is a valuable approach to understanding the complex dynamics underlying transmission of* Giardia* infections. In our study, assemblage B turned out to be the predominant genetical group in Cuban school children, but also a high frequency of mixed infections was found. Omission of washing hands before eating and keeping dogs at home were identified as significant risk factors of acquiring a* G. duodenalis* infection. In order to verify the possibility of zoonotic transmission and the potential of household pets as reservoir hosts, further large-scale studies involving microscopical coprology and PCR-based, multilocus genotyping of* Giardia* isolates in respective faecal samples are recommended and to get a few sequences combined with a phylogenetic analysis to see how the Cuban isolates are related to* Giardia* isolates identified in other places in the world. These investigations are expected to provide in future studies definite evidence for zoonotic transmission in giardiasis.

## Figures and Tables

**Table 1:** Characteristics of children infected with *G. duodenalis* in this study.

Characteristic	Number examined (*n* = 639)	Positive for *G. duodenalis* (*n* = 76) (%)	*P* value	Symptomatic children (*n* = 41) (%)	Asymptomatic children (*n* = 35) (%)	*P* value
Sex						
Male	357	42 (11.8)	0.92	23 (54.8)	19 (45.2)	0.87
Female	282	34 (12.1)		18 (52.9)	16 (47.1)	
Residing area						
Urban	558	69 (12.4)	0.38	38 (55.1)	31 (44.9)	0.54
Rural	81	7 (8.6)		3 (42.9)	4 (57.1)	
Colour of skin						
White	432	48 (11.1)		25 (52.1)	23 (47.9)	
Black	58	3 (5.2)		1 (33.3)	2 (66.7)	
Mestizo	149	23 (15.4)	0.32	15 (65.2)	8 (34.8)	0.43
Age						
<5	162	27 (14.1)		13 (48.1)	14 (51.9)	
>5	477	49 (10.3)	0.06	28 (57.1)	21 (42.9)	0.45
Diarrhea						
Yes	123	18 (14.6)		12 (66.7)	6 (33.3)	
No	516	58 (11.2)	0.36	29 (50.0)	29 (50.0)	0.22

**Table 2:** Distribution of assemblages and mixed infections among 76 DNA samples.

Type of PCR	Assemblage			Result	
	A (% from total positive)	B (% from total positive)	A + B (% from total positive)	Total positive	Not amplified
PCR-*tpi*	7 (11.1)	30 (47.6)	26 (41.3)	63	13
PCR-*E1-HP*	5 (8.6)	39 (67.3)	14 (24.1)	58	18

**Table 3:** Distribution of assemblages of *Giardia duodenalis *and association with clinical variables in all children carrying genotypes consistently identified in both PCRs.

Clinical data	Assemblages by PCR-*tpi* and PCR-*E1-HP*			*P* value (B versus A + B)
	B (*n* = 28) *n* (%)	A (*n* = 4) *n* (%)	A + B (*n* = 11) *n* (%)	
Asymptomatic	13 (46.4)	2 (50.0)	5 (45.4)	0.99
Symptomatic	15 (53.6)	2 (50.0)	6 (55.6)	

Diarrhoea	11 (39.3)	0 (0)	1 (9.1)	0.02^*∗*^
Abdominal pain	12 (42.9)	1 (25.0)	2 (18.2)	0.12
Flatulence	5 (17.9)	0 (0)	2 (18.2)	0.63
Nausea	6 (21.4)	1 (25.0)	4 (36.4)	0.53
Vomiting	5 (17.9)	1 (25.0)	2 (18.2)	0.90
Anorexia	6 (21.4)	1 (25.0)	3 (27.3)	0.90
Fever	5 (17.9)	1 (25.0)	1 (9.1)	0.63
Loss of weight	11 (39.3)	1 (25.0)	2 (18.2)	0.22
Fatigue	4 (7.1)	0 (0)	1 (9.1)	0.94

^*∗*^Statistically significant difference (B versus A + B).

**Table 4:** Analysis of potential risk factors associated with *G. duodenalis* infection among the children that participated in the present study.

Characteristics	* G. duodenalis*		* *Univariate		* *Multivariate	
	Negative	Positive *n* (%)	Odds ratio (95% CI)	*P* value	Odds ratio (95% CI)	*P* value
Other family members infected with giardiasis						
Yes	58	11 (18.9)	1.7 (0.85–3.39)	0.13	1.4 (0.63–2.27)	0.09
No	581	65 (11.2)	1		1	
Source of drinking water						
Safe water (chlorine-treated)	601	75 (12.5)	1		1	
Unsafe water (untreated)	38	1 (2.6)	0.2 (0.03–1.56)	0.09	0.4 (0.12–1.89)	0.17
Water boiled before consumption						
Yes	457	34 (7.4)	1		1	
No	182	42 (13.5)	1.4 (0.75–2.7)	0.27	1.1 (0.56–2.01)	0.34
Washing hands before eating						
Yes	338	31 (9.7)	1		1	
No	301	45 (23.1)	1.6 (1.00–2.64)	0.04^*∗*^	1.3 (0.89–2.23)	0.06
Washing hands after defecation						
Yes	621	74 (11.9)	1		1	
No	18	2 (11.1)	1.1 (0.24–4.71)	0.92	0.9 (0.38–3.91)	0.79
Washing vegetables/fruits before consumption						
Yes	498	47 (9.4)	1		1	
No	141	19 (13.5)	1.2 (0.68–2.04)	0.56	1.3 (0.80–1.97)	0.61
Keeping dog(s) at home						
Yes	71	33 (46.5)	2.6 (1.23–5.36)	0.004^*∗*^	2.1 (1.11–4.76)	0.01^*∗*^
No	76	12 (15.8)	1		1	

^*∗*^Statistically significant difference.
